# Complete Stone Clearance after Retrograde Intrarenal Surgery among Patients with Urolithiasis in a Tertiary Care Centre: A Descriptive Cross-sectional Study

**DOI:** 10.31729/jnma.7785

**Published:** 2022-12-31

**Authors:** Dipak Kumar Thakur, Chandra Sekhar Agrawal

**Affiliations:** 1 Department of Urology, Birat Medical College Teaching Hospital, Biratnagar, Morang, Nepal

**Keywords:** *laser*, *miniaturization*, *postoperative complications*

## Abstract

**Introduction:**

Retrograde intra-renal surgery using flexible scopes and laser energy is a newer alternative in stone disease treatment armamentarium. It is claimed to be superior to other modalities for stone clearance, complications and hospital stay. The aim of this study was to find out the prevalence of complete stone clearance after retrograde intra-renal surgery among patients with urolithiasis in a tertiary care centre.

**Methods:**

This was a descriptive cross-sectional study conducted in the Department of Urology in a tertiary care centre from 15 June 2021 to 14 May 2022 including adult patients with stone size up to 15 mm. Ethical approval was obtained from the Institutional Review Committee (Reference number: IRC-PA-143/2077-78). Convenience sampling was done. The prevalence of complete stone clearance (no residual fragment >4 mm) was calculated. Point estimation and 95% confidence interval were calculated.

**Results:**

Among 42 patients, 36 (85.71%) patients (75.1-96.3, 95% Confidence Interval) achieved complete stone clearance. The mean age was 40.26±14.05 (16-74) years and the stone size was 1.27±0.19 (0.9-1.5) cm. Similarly, the mean operating time was 51.55±9.34 (40-85) minutes and the hospital stay was 1.33±0.52 (1-3) days. Grade 3 ureteric injury occurred in one case. Residual fragments were seen in 6 cases (14.29%). Sepsis occurred in 4 cases (11.11%).

**Conclusions:**

The prevalence of complete stone clearance was similar among patients undergoing retrograde intra-renal surgery in our study when compared to other studies conducted in similar settings.

## INTRODUCTION

Retrograde intra-renal surgery (RIRS) using flexible scopes and laser energy is a newer alternative in stone disease treatment armamentarium, primarily used for stones <2 cm and its' use increasing as a preferred modality for larger stones (>2.5 cm).^12^ It is claimed to be superior to other modalities for morbidity, and hospital stays along with a high stone-free rate and minimal complications but is limited by its' delicate instruments, the cost factor and the long learning curve.^1,3-5^

In our institute, the RIRS facility is available and performed on a routine basis. However, to the best of our knowledge, the prevalence of complete stone clearance of this procedure has not been studied till date here.

This study aims to find out the prevalence of complete stone clearance after retrograde intra-renal surgery among patients with urolithiasis in a tertiary care centre.

## METHODS

This was a descriptive cross-sectional study conducted in the Department of Urology of Birat Medical College and Teaching Hospital over a period of one year from 15 June 2021 to 14 May 2022. The study was approved by the Institutional Review Committee (Reference number: IRC-PA-143/2077-78). Adult patients with stone sizes up to 15 mm who were planned for RIRS were included in this study. We excluded patients with active urinary infection, coagulopathy, stone size >15 mm and patients not consenting to the staged procedure. Convenience sampling technique was used.

The sample size was calculated using the following formula:


$$\begin{array}{ll} \text{n} & ={\text{Z}}^{2}\times \frac{\text{p}\times \text{q}}{{\text{e}}^{\text{2}}}\\ & =1.96^{2}\times \frac{0.94\times 0.06}{0.1^{2}}\\ & =22 \end{array}$$

Where,

n = minimum required sample size
Z = 1.96 at 95% Confidence Interval (CI)
p = prevalence of stone clearancetaken from previous study, 94%^6^
q = 1-p
e = margin of error, 10%

The minimum required sample size was 22. The final sample size taken was 42.

Patients were evaluated preoperatively as per institutional protocol. No patient was presented preoperatively and they received prophylactic antibiotic Inj. ceftriaxone 50 mg/kg and Inj. amikacin 15 mg/kg. The procedure was done either in general or with spinal anaesthesia. Semirigid ureteroscopy using 6.5/7 Fr ureteroscope from Karl Storz was done in all patients before the introduction of ureteral access sheath (UAS) [10.7/12.7 Fr, Cook Medical] or flexible scope (Lithovue, Boston Scientific). Patients who accommodated UAS underwent RIRS and those who did not accommodate UAS and consented to the staged procedure were stented in the setting and called after 2 weeks for RIRS. Calculus was dusted, fragmented or popcorned using a 20W LASER machine (Versa Pulse P20, Lumenis) and 200-micron laser fibre with energy and frequency range of 0.5-1.2 J and 6-14 Hz respectively.

At the end of the procedure, 6 F 26 cm DJ stenting was done in all patients and they received antibiotics, Proton pump inhibitor, analgesics (Inj. ketorolac 30 mg IV SOS) and alpha-blocker (tamsulosin 0.4 mg PO HS). X-ray KUB was done the next morning & patients were discharged on an oral antibiotic (tab. cefixime 200 mg BD), proton pump inhibitor, analgesics and alphablocker unless any complication occurred. Patients' demography, stone status, operating time, hospital stay and complications were recorded. DJ stent removal was done at 2 weeks postoperatively. Stone clearance was assessed at one month via Ultrasound, any residual fragment >4 mm was considered significant (CSRF).

Statistical analysis was performed using IBM SPSS Statistics version 27.0. Point estimate and 95% CI were calculated.

## RESULTS

Among the 42 patients, 36 (85.71%) patients (75.1396.29, 95% CI) achieved complete stone clearance at 1 month. Mean age of the patients was 40.26±14.05 with the range of 16-74 years with male predominance 21 (59.50%). Stones were equally distributed on both sides (50% each) and mean stone size was 1.27±0.19 (range, 0.9-1.5) cm. The location of the stone was pelvi-ureteric junction (PUJ)/ pelvis 18 (50%), upper calyx 8 (22.22%), proximal ureter 5 (13.89%), mid calyx 3 (8.33%) and lower calyx 2 (5.56%) respectively (Table 1).

**Table 1:** Baseline characteristics (n= 36).

Variables		n (%)
Gender	Male	21 (58.33)
	Female	15 (41.67)
Stone side	Right	18 (50)
	Left	18 (50)
Stone location	PUJ/pelvis	18 (50)
	Upper calyx	8 (22.22)
	Proximal ureter	5 (13.89)
	Mid calyx	3 (8.33)
	Lower calyx	2 (5.56)

Twenty-four patients (57.14%) underwent RIRS in the same settings while 18 patients (42.85%) underwent staged procedure. The mean operating time was 51.55±9.34 (range, 40-85) minutes. Similarly, the hospital stay was 1.33±0.52 (range, 1-3) days. Grade 3 ureteric injury occurred in one patient (2.38%) of impacted calculus at PUJ and required antegrade stenting. Urosepsis occurred in 4 (9.52%) cases which required early stent removal (within a week post procedure).

At the end of one month, stone clearance was achieved in 36 (85.71%) patients and 6 (14.29%) had clinically significant residual fragments (CSRF). They opted for conservative management and stone clearance was achieved during follow-up at the 3 months (Table 2).

**Table 2:** Postoperative characteristics (n = 36).

Complications	n (%)
Grade 3 ureteric injury	1 (2.78)
Sepsis	4 (11.11)
None	31 (86.11)

## DISCUSSION

The prevalence of complete stone clearance at one month was 85.71% in this study. It is better than the other similar studies done in Turkey, Nepal and Malaysia respectively with their stone clearance of about 70%.^7-9^ Our stone clearance was comparable with the results of another study done in Turkey which reported stone clearance of 87%.^10^ But our stone clearance was lower than a study done in India where 94.30% stone clearance was achieved. However, this result was reported at 3 months of follow up.^6^ These differences can be due to the nature of the case selection and generations of the flexible scopes and LASER machines used affecting the outcome of the procedure.

In the current study, 57% of patients accommodated UAS without prior stenting. Similarly, 94.50% of the cases accommodated UAS in cases which were not presented in a study done in India and in 68% of cases of a study conducted in Nepal indicating presenting is not mandatory before RIRS.^6,11^ Comparatively lower figure in our study can explained by selection of larger UAS here.

The calculus size in this study ranged from 0.9 to 1.5 cm which is comparable with the calculus size in the similar studies where it was 0.8 to 2 cm.^7,8,10^ In this study, the mean operating time was 51.5±9.3 minutes and hospital stay ranged from 1-3 days. Our results are comparable with other studies where the operating time ranged from 43 to 68 minutes and hospital stay 1-3 days respectively.^7,8,10^

Complications following RIRS vary from 0-25% and often they are minor.^12,13^ In our study, complication occurred in 5 cases (13.89%) which is comparable with the reported rate. The incidence of ureteric perforation following RIRS is estimated to be 0.9%-9.4%.^14,15^ One case (2.78%) in this study had grade 3 ureteric injury. It was a case of impacted PUJ calculus where in situ lasering was done. It required antegrade stenting.

Our study is limited by being a single-centre study with fewer patients. We did not calculate the stone-free rate according to the location of the calculus, volume and stone density because of the study design which can be a further limitation of our study. We recommend multicentric studies with a larger number of patients considering other parameters of interest.

## CONCLUSIONS

The prevalence of complete stone clearance was similar among patients undergoing retrograde intrarenal surgery in our study when compared to other studies conducted in similar settings.
